# Efficient U-Net Architecture with Multiple Encoders and Attention Mechanism Decoders for Brain Tumor Segmentation

**DOI:** 10.3390/diagnostics13050872

**Published:** 2023-02-24

**Authors:** Ilyasse Aboussaleh, Jamal Riffi, Khalid El Fazazy, Mohamed Adnane Mahraz, Hamid Tairi

**Affiliations:** Laboratory of Computer Science, Signals, Automation and Cognitivism (LISAC), Department of Computer Science, Faculty of Sciences Dhar El Mahraz, Sidi Mohamed Ben Abdellah University, Fez 30000, Morocco

**Keywords:** brain tumor segmentation, deep learning, U-Net, encoder, pyramid neural network, transfer learning, attention

## Abstract

The brain is the center of human control and communication. Hence, it is very important to protect it and provide ideal conditions for it to function. Brain cancer remains one of the leading causes of death in the world, and the detection of malignant brain tumors is a priority in medical image segmentation. The brain tumor segmentation task aims to identify the pixels that belong to the abnormal areas when compared to normal tissue. Deep learning has shown in recent years its power to solve this problem, especially the U-Net-like architectures. In this paper, we proposed an efficient U-Net architecture with three different encoders: VGG-19, ResNet50, and MobileNetV2. This is based on transfer learning followed by a bidirectional features pyramid network applied to each encoder to obtain more spatial pertinent features. Then, we fused the feature maps extracted from the output of each network and merged them into our decoder with an attention mechanism. The method was evaluated on the BraTS 2020 dataset to segment the different types of tumors and the results show a good performance in terms of dice similarity, with coefficients of 0.8741, 0.8069, and 0.7033 for the whole tumor, core tumor, and enhancing tumor, respectively.

## 1. Introduction

Brain tumors account for 85% to 90% of all primary central nervous system (CNS) tumors. Worldwide, an estimated 308,102 people were diagnosed with a primary brain or spinal cord tumor in 2020. Two years later, the number increased to 700,000 in the United States, and approximately 88,970 more will be diagnosed according to the national brain tumor society (NBTS). Globally, over 241,000 die each year because of brain tumors or nervous system cancer and each year the number of people who die increases. Glioma is one of the most common types of brain tumor and is also known as a primary brain tumor. Although the exact origin of gliomas is still unknown, there are two grades of glioma: low-grade glioma (LGG) and high-grade glioma (HGG). The latter is the most aggressive and very infiltrative because it quickly spreads into other parts of the brain; thus, then early detection of the tumor is very crucial because it enhances the rate of survival and facilitates the therapy phase.

**Figure 1:**
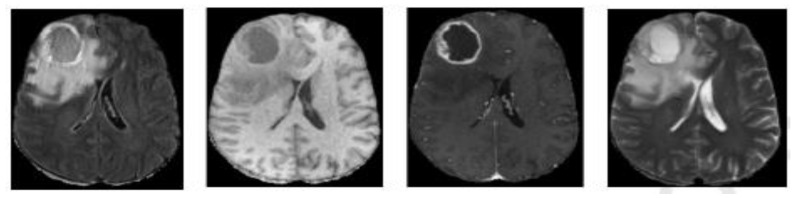
Different modalities of an MRI image from left to right: Flair, T1, T1ce, and T2.

Medical imaging analysis comes to help patients and saves people’s lives by diagnosis using new safety technology, such as positron emission tomography (PET), computed tomography (CT), and magnetic resonance imaging (MRI). T1-weighted, T2-weighted, T1-weighted with contrast enhancement (T1ce), and fluid-attenuated inversion recovery (FLAIR) are the four modalities of MRI images, as seen in Figure 1, and each one is in 2D slices form and puts all the slices together produce a 3D form of the brain. Utilization of multiple modalities and sequences to segment the brain tumor can improve results and provide complementary features on regions of different sub-gliomas. Semi-automatic and automatic approaches have been proposed in the brain tumor segmentation area and the automatic one showed its performance and a high potential for more accurate and reliable results.

Therefore, numerous studies have proved to detect and segment different types of brain tumors without using ground truth labels. Based on machine learning (ML) algorithms, K-means clustering is frequently used to separate an interest region from an image. K-means has undergone thorough testing in the segmentation of brain tumors and has demonstrated acceptable accuracy [1,2]. Almahfud et al. [3] proposed a combination of K-Means and Fuzzy C-Means. They applied this combination to make the image more visible. Then, they mapped it, applied a median filter, and used morphological area selection to eliminate small pixels and detect the location of the tumor [4]. A genetic algorithm is relied on to create a new technique of segmentation discrete wavelet transform and a fitness function variance as an objective function. This method obtained a high performance in terms of accuracy. 

For supervised approaches with ML, Cui et al. [5] extracted features using an intensity texture after image registration in the preprocessing phase. Multi-kernel support vector machine (SVM) is employed as a classifier and a region growing to postprocess the results. Chen et al. [6] used N4ITK, histogram matching, and simple linear iterative clustering for preprocessing, gray statistical and gray-level co-occurrence matrix for feature extracting, and SVM as a classifier [7,8]. They employed other classifiers, random forest, morphological techniques, and some filtering methods in postprocessing to segment tumors. Therefore, the first used noise removal in preprocessing and the first higher-order plus texture as a vector of features, and the second was based on histogram enhancement and Gabor wavelet in addition to intensity in preprocessing and feature extracting, respectively.

The intensity non-uniformity in MRI imaging makes the feature’s extracted phase more complex in ML methods, and the amount of this type of data affects the performance of most ML algorithms and limits their results. Deep learning comes to solve this type of limitation and it has proven its performance in medical imaging analysis and retrieval [9,10] in general, and in medical imaging segmentation specifically. Convolution neural networks (CNNs) and the encoder–decoder with skip connection is the first and the most used in this area. Therefore, Pereira et al. [11] employed a custom CNN followed by bias field correction, intensity, patch normalization, and data augmentation. The methods [12,13] integrated a full CNN to segment different regions of the tumor, and then [12] FCNN was combined with conditional random forest (CRF). On the other hand [13], a cascade of FCNN is proposed to decompose the multi-classes segmentation problem into three binary segmentations. 

Aboussaleh et al. [14] used the features extracted from the last convolution layer of a CNN-proposed model, calculated a gradient of those features, stocked the mean and the max of each one in two vectors, and multiplied them by the features component by component. Finally, a thresholding and morphological process to postprocess the whole tumor was used. This method did not use the mask, but it obtained a high performance in terms of dice coefficient similarity. On the other hand, U-Net-like architectures showed their majority and success. U-Net is a symmetric fully convolutional network proposed by Ronneberger et al. [15] with a decoder path to ensure precise position and an encoder path to capture context information. U-Net is still used as a reference in both 2D and 3D brain tumor segmentation, and several methods were inspired by making adjustments to the encoder, skip connection, or decoder parts. Liu et al. [16] proposed a novel cascade U-Net in which each basic block is designed as a residual one to overcome the vanishing gradient problem. Additionally, they designed some skip connections to enhance the features transmitted between the encoder and decoder. Aboelenein et al. [17] introduced a hybrid two-track U-Net. They merged two tracks, and each one employs a different kernel and number of layers to obtain a final segmentation result. The architecture employed batch normalization and it chose Leaky *ReLU* as an activation function. Recently, U-Net has been combined with transfer learning in the latest research to solve a complex limitation of contraction path in U-Net. A lot of time is spent on its execution using a pre-trained model and obtaining more significant features. Moreover, U-Net-VGG16 [18] was one of those contributions. Then, they replaced the encoder path with VGGNet [19]. The same idea was applied to several hybrid architectures replacing VGG-Net with other CNN architectures, such as LeNet [20], AlexNet [21], MobileNet [22], and ResNet [23]. Meanwhile, these methods still raise challenges to learning global semantic information, which is critical for segmentation tasks; therefore, the attention mechanism was introduced to overcome these challenges. 

Fusing CNN-based methods, U-Net architectures and attention mechanisms can allow for extracting more precise dense feature information in the downsampling, and they can effectively recover spatial information and position details in the upsampling path. In this context, Zhang et al. [24] proposed Attention Gate ResU-Net for automatic MRI brain tumor segmentation. They employed a residual block and an attention gate with a single U-Net architecture added into the skip connection part. On the other hand, Wu et al. [25] developed a new method based on generative adversarial network (GAN) named symmetric driven GAN. The method was trained and learned a non-linear mapping betwixt the left and right brain images, along with the variability of the brains. 

Another method that relies on GAN has been proposed by Dey et al. [26]. They introduced a framework named the Adversarial-based Selective Network ASC-Net that aims to decompose an image into two selective cuts based on a reference image distribution. One cut will fall into the reference distribution, while other image content outside of the reference image distribution will group into the other cut. These two cuts reconstruct the original input image semantically and apply simple thresholding to regroup normal and abnormal regions.

In this paper, we developed a new architecture belonging to U-Net-like ones. The architecture consists of two parts: an encoder and a decoder. The first part used three different pre-trained models of CNNs to create a multiple encoder in order to extract more local features. We introduced the features extracted from each encoder as input into a bidirectional feature pyramid network (Bi-FPN) to enrich them, and a concatenation has been affected into those Bi-FPN outputs to obtain overall specific features. In the second part, we upsampled the encoded feature map based on the attention mechanism that allows us to better preserve fine details and ignore irrelevant information about those features and to produce a segmentation mask that is the same size as the input image. Section 2 will describe the materials and methods and Section 3 will be devoted to representing the results. Then, Section 4 is mainly concerned with discussion and conclusions.

## 2. Materials and Method

### 2.1. Data and Data Preparation

#### 2.1.1. Dataset

The BraTS 2020 [27,28,29] contest provides a large training set of 369 MRI scans and a validation set of 125 scans. Each scan was 240 × 240 × 155 in size, and each case had FLAIR, T1, T1 extension, and T2 volumes. The dataset is co-registered, re-sampled to $1\times 1\times 1{\;\mathrm{mm}}^{3}$, and skull-stripped. Segmented brain tumors include necrosis, edema, non-enhancing, and enhancing tumors. The ground truth of the training set was only obtained by manual segmentation results given by experts.

#### 2.1.2. Data Preparation

**Figure 2:**
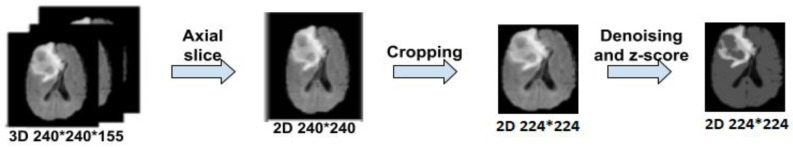
Overall steps of brain tumor data preparation.

BraTS is a 3D dataset, and since our proposed architecture relies on 2D images, we transformed each patient’s size from 240×240×155 to 240×240 by choosing the middle slice of each modality, cropped it to 224×224 to eliminate some insignificant background pixels, and applied Gaussian denoising, as seen in Figure 2. The z-score normalization was performed by subtracting the mean µ of the input image i and divided by its standard deviation σ to obtain $i_{0}$, as Equation (1) demonstrated. Data augmentation was applied to our data by simple transformation, such as flipping, rotating, adding noise, and translating.

(1) $$i_{0}=\frac{i-µ}{\sigma \;}$$

### 2.2. Methods

**Figure 3:**
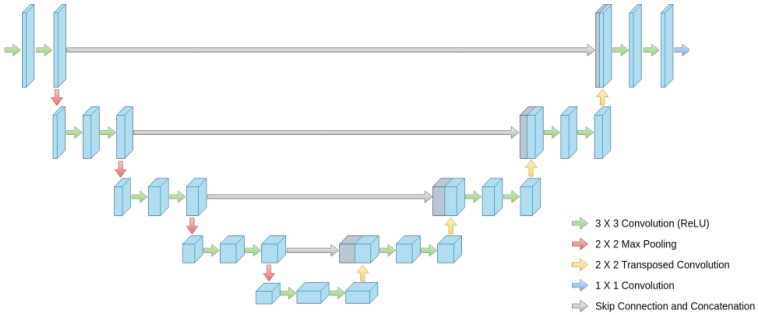
Global architecture of U-Net.

The model architecture takes inspiration from the U-Net architecture represented in Figure 3 to create a new enhanced model for brain tumor segmentation.

We use three pre-trained models VGG-19, MobileNetV2 and ResNet50 in the encoder part, deleting the layers of the classification stage and using fine tuning to retrain the weights of all the convolution and pooling layers. Each pre-trained model takes as input one slice (middle one) from the 155 that are possible and obtains an output feature at five corresponding depths, which are the respective inputs of the Bi-FPN. Bi-FPN is an enricher-features employed used in Efficient-Det.

**Figure 4:**
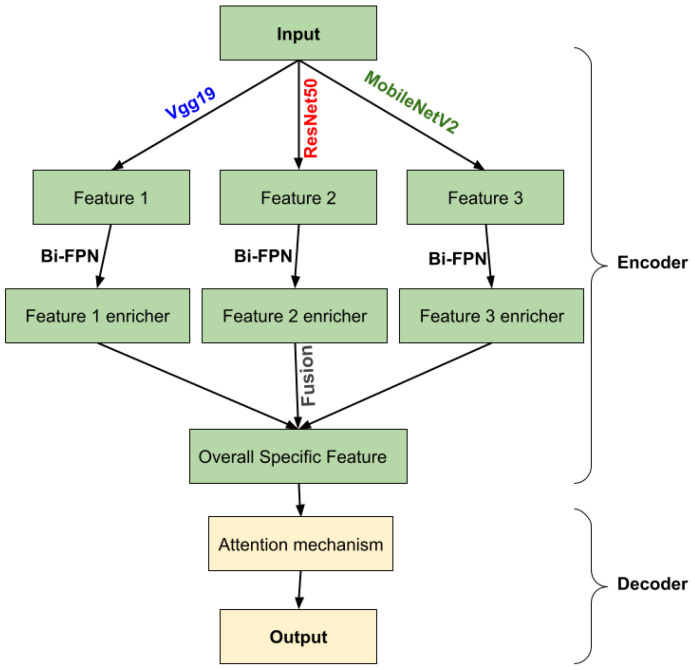
Overview of the proposed methodology.

The feature network’s outputs are combined into a decoder stage. In this stage, we calculate the gating signal and make it as input with feature extraction in the encoder part into an attention block, performing the same process for each depth, and finally an output convolution block to obtain the brain tumor segmentation. Figure 4 illustrates an overview of the proposed method.

#### 2.2.1. Encoder

##### Transfer Learning

Transfer learning is an approach for starting computer vision and language processing tasks with pre-trained models by applying the knowledge from the source task to the work at hand. Transfer learning seeks to enhance learning in the target task. It is a viable technique for minimizing learning time. This technique might be connected to creating deep learning models for image classification problems. Based on the ImageNet dataset which contains more than 1.2 million images and 1000 targets, VGGNet19, MobileNetV2, and ResNet50 are three of several pre-trained models used in classification. We employed them in our encoder part by eliminating the classification stage (i.e., the fully connected layers) since we need the output of the last layer of each convolution block the extraction features stage (i.e., the convolutional and pooling layers). All these outputs will be used as input to a Bi-FPN to extract more features. Fine tuning was applied to retrain all the weights in order to adapt them to our segmentation problem.

- *VGG-19*

**Figure 5:**
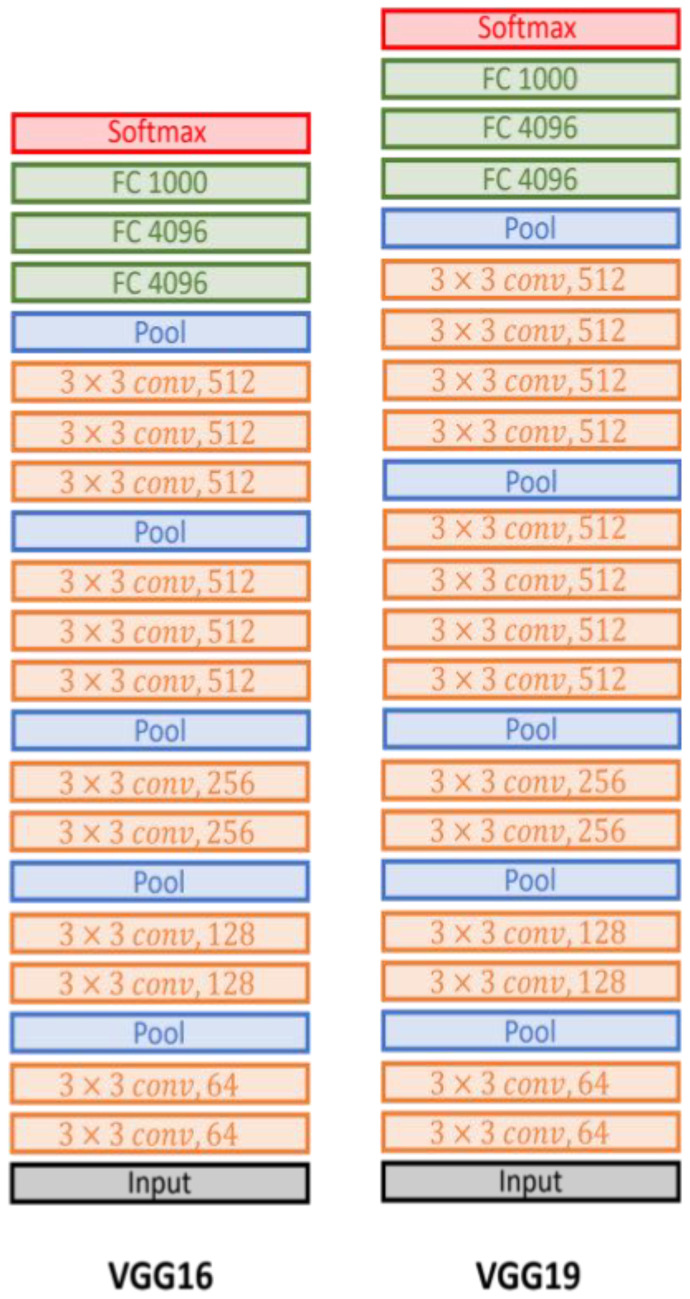
The VGG-16 and VGG-19 architectures.

The VGG network, or VGGNet, is a deep neural network architecture. Its contribution is proving that the depth of the network is a critical component to achieving better recognition or classification accuracy in CNNs. The VGG network is constructed with very small 3×3 filters. The reasoning behind the usage of 3 **×** 3 filters by VGGNet is that three 3 **×** 3 filters provide a receptive field of 7 × 7 filters, and two consecutive 3 × 3 filters provide a 5 × 5 effective receptive field. The number of filters doubles after every max-pooling operation. VGG-16 and VGG-19 are illustrated in detail in Figure 5. The only difference was in the number of layers because the first one used 16 layers and the second increased the number to 19.

- *ResNet50*

**Figure 6:**
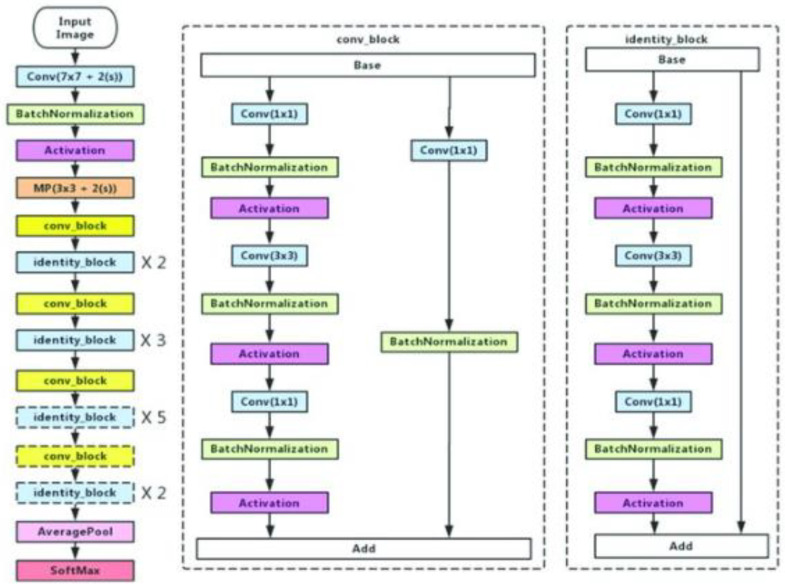
ResNet50 architecture.

Residual networks, or ResNet50, is a variant of the ResNet model which has 48 Convolution layers along with 1 MaxPool and 1 Average Pool layer. ResNet is built of a residual block, which is shown in Figure 6, by stacking residual blocks together, and each residual block has two 3 × 3 convolution layers Periodically, we doubled the number of filters and downsampled using stride 2. The ResNet does not have fully connected layers to output the 1000 classes.

- *MobileNetV2*

**Figure 7:**
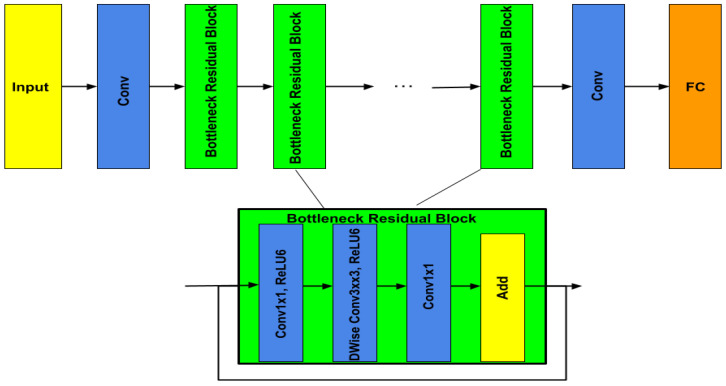
MobileNetV2 architecture.

MobileNetV2, illustrated in Figure 7, is a new version of MobileNetV1 [30]. Therefore, MobileNetV1 is based on depthwise separable convolution in the first layer to reduce the complexity cost and model size of the network, and a 1×1 convolution in the second layer was used for building new features through computing linear combinations of the input channels. On the other hand, MobileNetV2 used two types of blocks. One is a residual block with a stride of 1, and the other one is a block with a stride of 2 for downsizing. They employed 3 layers for both types of blocks, but they started with the layer of 1×1 convolution with *ReLU*6. After a layer of depthwise convolution was applied, the last layer was 1×1 convolution but without any non-linearity.

(2) ReLU6=min(max(x,0),6)

##### Bi-Directional Feature Pyramid Network (Bi-FPN)

**Figure 8:**
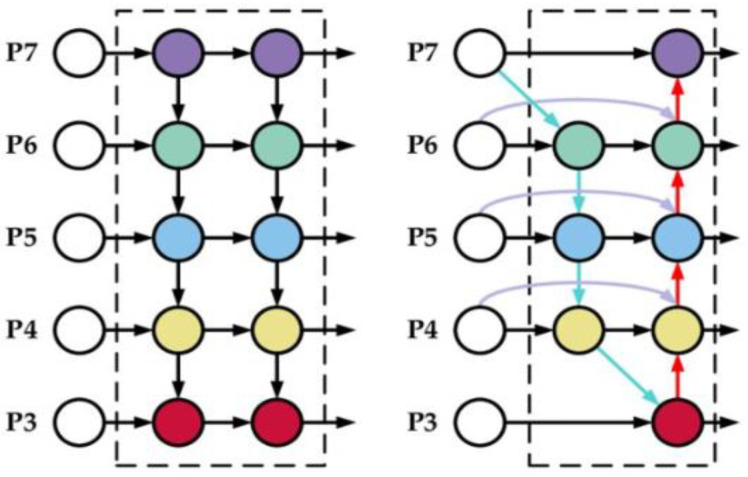
Feature design, **left**: FPN, **right**: Bi-FPN.

The Bi-FPN is based on the traditional top-down feature pyramid network (FPN), as seen in Figure 8, developed in 2017 by Lin et al. [31]. It takes level 3–7 input features ${\vec{p}}^{in}=\left(p_{3}^{in},\ldots \ldots \ldots ,p_{7}^{in}\right)$ where $p_{i}^{in}$ represents a feature level with a resolution of $1/2^{i}$ for the input. The conventional top-down FPN aggregates multi-scale features in a top-down manner:

(3) $$\begin{array}{l} p_{7}^{out}=Conv\left(p_{7}^{in}\right)\\ p_{6}^{out}=Conv\left(p_{6}^{in}+Resize\left(p_{7}^{out}\right)\right)\\ \ldots \ldots \\ p_{3}^{out}=Conv\left(p_{3}^{in}+Resize\left(p_{4}^{out}\right)\right) \end{array}$$

where Resize is usually upsampling and downsampling operation and Conv is usually a convolution operation for feature processing. Top-down FPN is inherently limited by the one-way information flow. To address this issue, BiFPN integrates bidirectional cross-scale connections [32,33,34]. The cross-scale connection’s intuition is a node that has one input edge with fusion features having more contribution than the input edge with no feature fusion, adding an extra edge from the original input to the output node if they are at the same level and treating each bidirectional (top-down and bottom-up) path as one feature network layer and repeats the same layer multiple times to enable more high-level feature fusion. Furthermore, a depthwise separable convolution was adopted [35] for feature fusion and batch normalization and activation were added after each convolution to further increase efficiency.

In our paper, the BiFPN takes as input a list of features extracted from the classification stage blocks of each pre-trained model. $f_{1}{}^{in},\;f_{2}{}^{in}and\;f_{3}{}^{in}$ where $f_{1}{}^{in}$ is the list of the first pretrained model VGG-19 features. $f_{2}{}^{in}$ is the list of ResNet50 features and $f_{3}{}^{in}$ is the list of MobileNetV2 features. Then, each list contains five layers $L_{1},\;L_{2},\;L_{3},\;L_{4}\;and\;L_{5}$. The output of this network will be a list of features, $f_{1}{}^{out},\;f_{2}{}^{out}and\;f_{3}{}^{out}$, such that each one corresponds to its input feature list. In another way, applying BiFPN to $f_{1}{}^{in}$ generates $f_{1}{}^{out}$, $f_{2}{}^{in}$ generates $f_{2}{}^{out}$, and $f_{3}{}^{in}$ generates $f_{3}{}^{out}$. The 5-level Bi-FPN layers employed in each pre-trained model made the output calculus as follows:

(4) $$\begin{array}{l} L_{4}{}^{mid}=Conv(L_{4}{}^{in}+Resize\left(L_{5}{}^{in})\right)\\ L_{3}{}^{mid}=Conv(L_{3}{}^{in}+Resize\left(L_{4}{}^{mid})\right)\\ L_{2}{}^{mid}=Conv(L_{2}{}^{in}+Resize\left(L_{3}{}^{mid})\right)\\ L_{1}{}^{out}=Conv(L_{1}{}^{in}+Resize\left(L_{2}{}^{mid})\right)\\ L_{2}{}^{out}=Conv(L_{2}{}^{in}+L_{2}{}^{mid}+Resize\left(L_{1}{}^{out})\right)\\ L_{3}{}^{out}=Conv(L_{3}{}^{in}+L_{3}{}^{mid}+Resize\left(L_{2}{}^{out})\right)\\ L_{4}{}^{out}=Conv(L_{4}{}^{in}+L_{4}{}^{mid}+Resize\left(L_{3}{}^{out})\right)\\ L_{5}{}^{out}=Conv(L_{5}{}^{in}+Resize\left(L_{4}{}^{out})\right) \end{array}$$

$L_{i}{}^{in}$ is an element of the list $f_{i}{}^{in}$ which contains the output of each depth’s pre-trained model. It will be the Bi-FPN input layer. $L_{i}{}^{mid}$ and $L_{i}{}^{out}$ are the middle and output Bi-FPN layers respectively. Finally, we obtained three lists of features $f_{1}{}^{out},\;f_{2}{}^{out}\mathrm{and}\;f_{3}{}^{out}$. These lists will be merged to obtain a global list of specific features that will act as the input for our decoder path.

**Figure 9:**
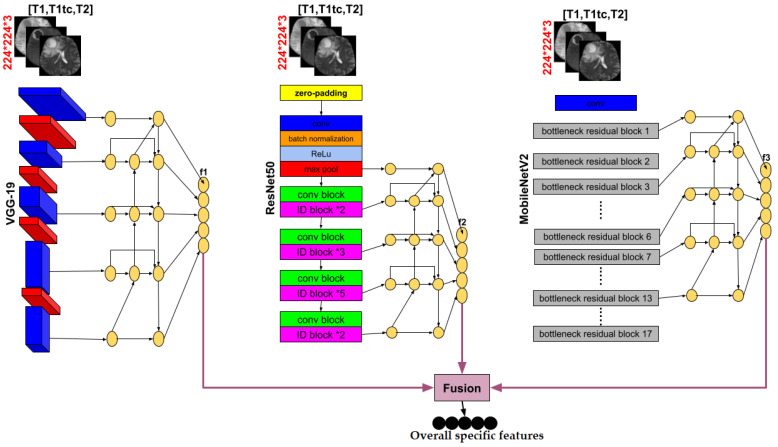
Details of the encoder part of our proposed method.

Our proposed encoder is represented with details in Figure 9.

#### 2.2.2. Decoder

**Figure 10:**
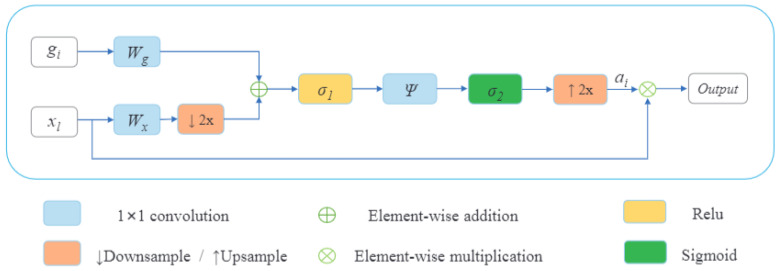
Schematic of the attention gate.

In this section, for each decoder layer *D*_i,_ each deconvolution block named UpAtt starts with a block of attention followed by an upsampling to increase the dimension and a double convolution block in the end. A double convolution block contains two convolution layers. It consists of a batch normalization layer and is activated by the activation function, *ReLU*. The last features obtained in global specific features will play the bottleneck role; then, four decoder layers will be obtained after each UpAtt, and a final output block that contains a convolution layer will be affected to obtain the segmented image with a different type of tumor. Adding an attention mechanism to our decoder generates layers containing more pertinent and deeper feature representation, and it pays attention to a small region of a brain tumor which improves the segmentation effect of brain tumors. Attention blocks or attention gates (AGs) are inspired by human mechanism attention which naturally concentrates on the region of interest and develops the ability to suppress unnecessary feature responses in feature maps while highlighting significant feature information critical for a specific task. The basic schematic of the attention gate is illustrated in Figure 10.

Where $x_{l}$ is the feature map of the l layer and $g_{i}$, is the gating signal vector used for each pixel i to select the focus regions on a coarser scale. The attention coefficient $\alpha_{i}$ belongs to the interval [0;1]. It identifies prominent image regions and curbs useless feature information to preserve only the activations relevant to the specific task. The AG output is the wise multiplication between the attention coefficient $\alpha_{i}$ and the feature map $x_{l}$.

(5) $$x_{output}=x_{l}\;\cdot \;\alpha_{i}$$

**Figure 11:**
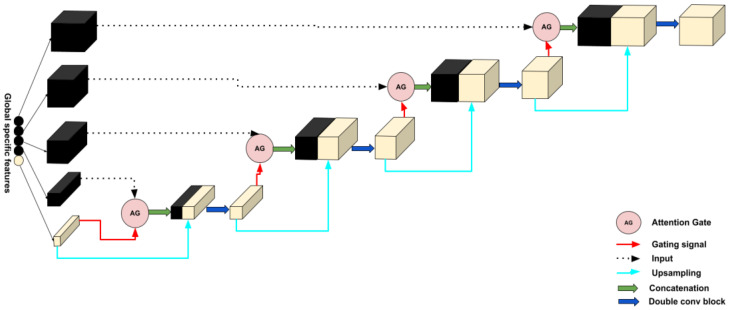
Details of the decoder part of our proposed method.

Brain tumor segmentation is a multiple semantic class task. Then, we employ a multi-dimensional attention coefficient [36] to focus on a subset of target regions. The multi-dimensional attention coefficient can be computed as:

(6) $$\alpha_{i}=\sigma_{2}\left(\psi^{T}\left(\sigma_{1}\left(W_{x}^{T}x_{l}+W_{g}^{T}g_{i}+b_{g}\right)\right)+b_{\psi }\right)$$

where $\sigma_{1}$ is defined as a *ReLU* function $\sigma_{1}\left(x\right)=\max\left(0,x\right)$ and $\sigma_{2}$ is the Sigmoid function. $\sigma_{2}\left(x\right)=\frac{1}{1+e^{-x}}$, $W_{x}$, $W_{g}$, and ψ are linear transformations, and $b_{g}$ and $b_{\psi }$ are biased terms. A 1×1 has been used as a channel-wise convolution for more performance to the linear transformation on the feature map $x_{l}$ and sigma gate $g_{i}$. Xavier normalization is employed to normalize parameters followed by the back-propagation algorithm to update weights. To continue our decoder path, we concatenate the AG output with the deconvoluted bottleneck and apply double convolution to this concatenation to obtain the output of our decoder block UpAtt. Figure 11 shows the details of our decoder’s proposed method.

$$\begin{array}{l} \;D_{1}=UpAtt\left(y^{bootlneck},AG^{out}\right)\\ D_{2}=UpAtt\left(D_{1},AG^{out}\right)\\ D_{3}=UpAtt\left(D_{2},AG^{out}\right)\\ D_{4}=UpAtt\left(D_{3},AG^{out}\right)\; \end{array}$$

A final block will be applied to the last decoder layer $D_{4}$ to obtain the final result. The block contains a convolution layer with four outputs. Each one corresponds, respectively, to the four classes defined as background, necrotic core, non-enhancing tumor peritumoral edema, and enhancing, followed by batch normalization and the SoftMax activation function.

**Figure 12:**
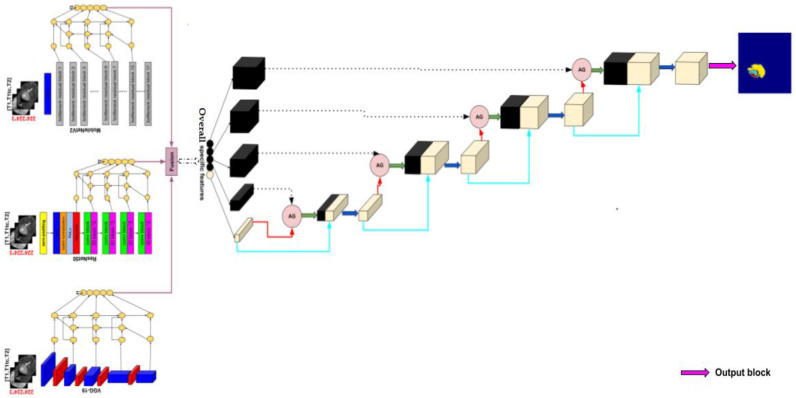
Proposed method architecture.

Figure 12 shows the encoder, decoder, and the image segmented, which resumes the proposed architecture, for brain tumor segmentation task. 

## 3. Results

In this section, we will present some implementation details of our model and cover the results obtained through our method based on some proposed evaluation metrics. 

### 3.1. Implementation Details

In this experiment, we used SIMPLTIK, a multidimensional open-source program Image analysis was performed with Python for image registration and segmentation to read MRI images from BraTS2020 data with the NIFTI format type. The experiment was carried out on the Kaggle platform in a virtual instance equipped with CPUs, 13GB memory, and an HDD drive of 73 GB. During the training of the model, acceleration was performed on Tesla (P100-PCIE-16GB) GPU (16GB video memory) and it takes 7 h to converge. The absence of a server with high performance makes our execution environment very limited and required optimized data by employing a lonely image from a 3D dataset to be able to execute our code in the Kaggle platform. The transfer learning used in our method forces us to have the number three as the number of channels in the input image size. This is why we must choose three sequences among the four possible (t1, t2, T1ce, and flair) for each input image, which makes the number of potential cases keeping the importance of order 24. For this, Kronberg et al. [37] proposed the best order to be carried out after comparing all the possible cases to the case or the absence of one or more sequences. From this article, the best recommended order we use is [t1, t1ce, t2]. Note that in each sequence, we chose the 90th slice of 155 (the slice when all the different types of tumors appear). The training dataset was divided randomly into the train, validation, and test subsets with 80:10:10 ratios. The parameters chosen for each pretrained model in the encoder part is explained in Section 2.2.1. For Bi-FPN networks, we employed a block of convolution with 32 kernels. The size of each one equals 1 and has a stride of 1. On the other hand, the stride of each upsampling and downsampling operation is 2. The block of depthwise convolution used after each resizing operation (upsampling and downsampling) employed a kernel size of 3 and a stride of 1. Table 1 shows the output of each encoder after applying the Bi-FPN networks before passing to the decoder part of the architecture. This last part is based on the attention mechanism. Next, we used in each depth of our decoder an attention block that takes as input the features obtained from the encoder and its corresponding gating signal and 128 as the number of kernels. This block is followed by an upsampling operation with a stride of 2 and a double convolution layer with 128 kernels with a size 3. The final convolution block, applied to obtain our output, employed 4 kernels with a size 1 and a SoftMax function activation. The loss function used for our model was the dice loss [38] which is used by computing the following average:

(7) $$Dice\left(P,G\right)=\frac{2\sum_{i=1}^{N}p_{i}g_{i}}{\sum_{i=1}^{N}p_{i}2+\sum_{i=1}^{N}g_{i}2}$$

where P represents the predicted value and G stands for the mask which represents the ground truth, $p_{i}\in P$ and $g_{i}\in G$. To minimize this loss function, we used an Adam optimizer with an initial learning rate of $\alpha_{0}=10^{-4}$ and progressively decreased it according to:

(8) $$\alpha =\alpha_{0}\times {\left(1-\frac{e}{N_{e}}\right)}^{0.9}$$

where e is an epoch counter and $N_{e}$ is the total number of epochs. In our case, the maximum number of epochs = 350 and in every epoch, the batch size = 5. Finally, a model checkpoint callback is used in conjunction with training to save the best weights of our model. 

### 3.2. Evaluation Metrics 

We have utilized various evaluation parameters to evaluate the performance of our proposed method, each of which is defined below:

- Accuracy: Formally, accuracy has the following definition:

$$Accuracy=\frac{TruePositive+TrueNegative}{Total}$$

- Precision: Formally, precision has the following definition:

$$Precision=\frac{TruePositive}{TruePositive+FalsePositive}$$

- Recall: Formally, recall has the following definition:

$$Recall=\frac{TruePositive}{TruePositive+FalseNegative}$$

- F1-score: Formally, F1-score has the following definition:

$$F1\;score=2\times \frac{Precision\times Recall}{Precision+Recall}$$

- The DSC represents the overlapping of predicted segmentation with the manually segmented output label and is computed as:

$$DSC=2\times \frac{\left|G\cap S\right|}{\left|G\right|+\left|S\right|}$$

- The IoU is used when calculating mean average precision (mAP). It specifies the amount of overlap between the predicted and ground truth, and it is computed as:

$$IoU=2\times \frac{Area\;of\;Overlap}{Area\;of\;Union}$$

- The Hausdorff95 distance measures the distance between the surface of the real area and the predicted area which is more sensitive to the segmented boundary defined as:

$$Haus95\left(T,\;P\right)=\max\left\{\underset{t\in T\;,\;p\in P\;}{\sup\;\inf}\;d\left(t,p\right),\;\underset{p\in P\;,\;t\in T\;}{\sup\;\inf}\;d\left(t,p\right)\right\}$$

where sup denotes the supremum, inf denotes the infimum, and t and p denote the points on the surface T of the ground truth area and the surface P of the predicted area. d (·,·) is a function of the distance between the points t and p. 

### 3.3. Results and Discussion

**Figure 13:**
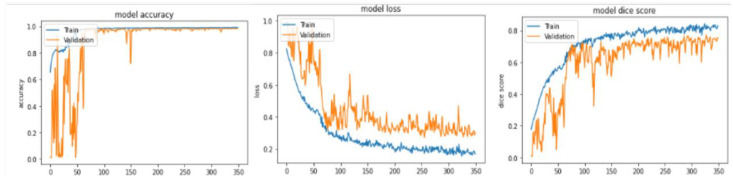
Accuracy, loss, and dice score of training and validation subsets in terms of the number of epochs from the BraTS 2020 training dataset.

In this subsection, we will discuss all the results obtained from our method, analyze them, compare them with some state of art methods, and visualize some qualitative results. To evaluate our model, we divided the BraTS 2020 training dataset into three subsets: training, validation, and test, with a ratio of 80:10:10 (295 for training, 37 for validation, and 37 for test). Table 2 and Table 3 show high performance in all metrics, especially in terms of the dice similarity coefficient of the whole tumor, Hausdorff95 distance of all the three types of tumors, precision, F1-score, recall, and accuracy for both subsets. Therefore, the proposed method achieved 87.89% and 78.39% of DSC and IoU of the whole tumor in the validation subset better than the DSC and IoU calculated from the test subset that achieved 87.89% and 77.64%, respectively. The evaluation metrics of the core tumor and enhancing tumor show their higher rank in comparison to validation ones, where they achieved 80.69% and 70.33% DSC of core tumor and enhancing tumor, respectively, 67.63% and 54.24% IoU of core tumor and enhancing tumor, respectively, 0 mm, 1 mm, and 0 mm of HD95 whole, core, and enhancing tumor, respectively. Good and acceptable results have been obtained in terms of precision, F1-score, and recall, where they all crossed the 83% and had a great accuracy of 99.77%, 99.23%, and 98.30% of the whole tumor, core tumor, and enhancing tumor, respectively. Figure 13 illustrates the curve of the accuracy, the loss, and the dice score of the training and validation subsets in terms of the number of epochs. The metrics converge after 350 epochs. To save memory and time, we stopped at this number regardless of the values initialized to the kernels.

To demonstrate the strength of our method, a comparison study has been conducted and showed in Table 4, between our proposed approach and some approaches from the state of art section and some others out of the state of the art. The unsupervised methods [3,14,25,39] in this comparison study are limited to calculating the metrics of the whole tumor because of the variation of the pixel’s intensities of each image in the BraTS 2020 dataset that makes the initialization of kernels and the choice of the corresponding thresholds a very hard task. This justifies the performance obtained from these methods, which yielded good results in comparison with the several methods that are not based on the labels (ground truth). The supervised methods [15,17,18,24,40,41,42] reach high results, especially those based on the U-Net architecture. Our approach exceeds all the others in terms of DSC that concerns the whole and the core tumor at 87.41% and 80.69%, respectively. On the other hand, HTTU-Net [17] obtains the best score of DSC in terms of the enhancing tumor equal to 80.80%. 

The main contribution employed in our method is very significant. It produces an efficient U-Net architecture that generates very important results. The combination of these three modifications (multiple encoders, BiFPN, and attention mechanisms) makes our U-Net more powerful. However, the omission of any of these modifications can negatively affect our method and degrade its results. Table 5 showed an ablation study of our method. Therefore, the results obtained when using one encoder (VGG-19, MobileNetV2, or ResNet50) with a simple decoder, containing an upsampling operation followed by concatenation and a convolution operation, are less than the results obtained when we employed the three different encoders and combined them after applying a BiFPN followed by a simple decoder. The ablation study demonstrated that the use of attention in the decoder phase, using a single encoder or multiple decoders, degrades the results. This shows the impact of BiFPN on the performance of our proposed approach.

**Figure 14:**
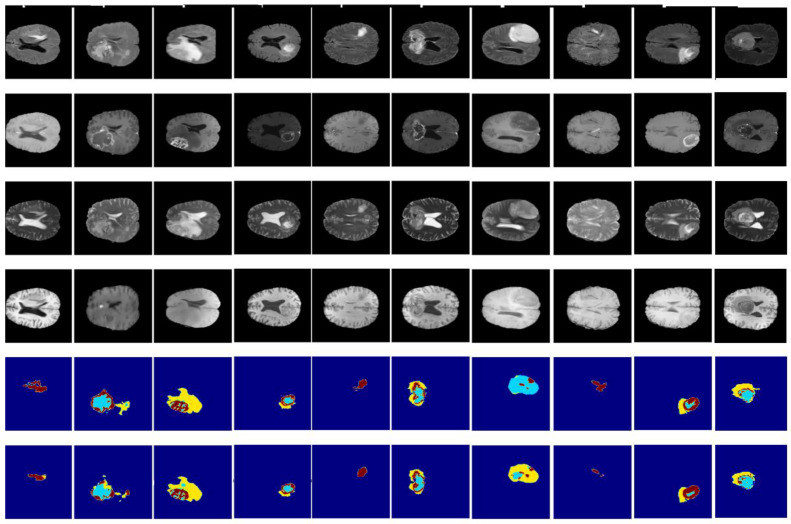
Qualitative results obtained from the validation subset of the BraTS 2020 training dataset. From up to down: flair, t1ce, t1, t2, ground truth, and prediction.

**Figure 15:**
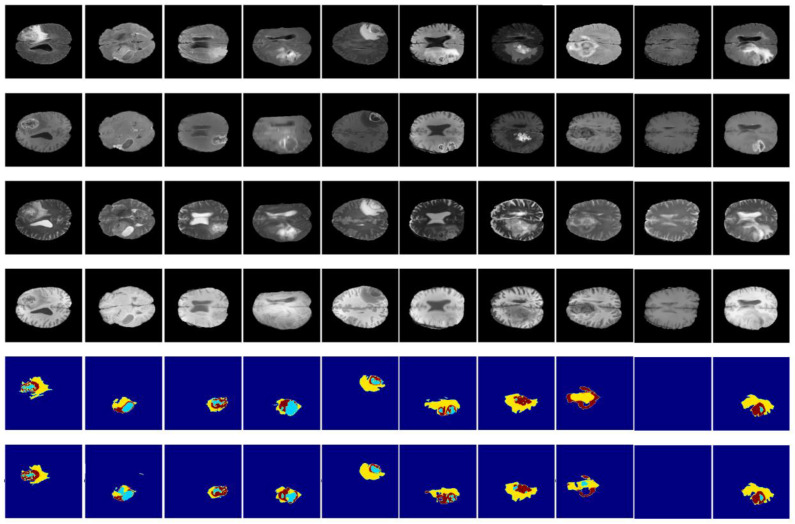
Qualitative results obtained from the test subset of the BraTS 2020 training dataset. From up to down: flair, t1ce, t1, t2, ground truth, and prediction.

Figure 14 and Figure 15 illustrate a qualitative result of our method from the validation and test subset, respectively. Globally, the whole tumor has been segmented very well and also the images without the tumor have a good result (no segmentation in the prediction images). In addition, the core tumor has been segmented in an acceptable way. Some images are good for visualization and others are not. Finally, many images of initial tumors are not well segmented. This last type of tumor needs improvement, which is our objective for future work.

## 4. Conclusions

In this paper, we proposed an efficient U-Net architecture specialized for brain tumor segmentation. Three main combinations made a new contribution and achieved a good performance based on different metrics. The encoder of our approach used three different pretrained models: VGG-19, MobileNetV2, and ResNet50, applying a BiFPN to each one to generate more spatial significant features before the fusion operation. At the decoder part, we employed the attention mechanism. This has proven itself in medical image analysis, especially in segmentation problems by focusing more on different types of tumors to facilitate the segmentation task. We have trained and evaluated our method on the BraTS2020 dataset using ground truths (extracted by medical experts), compared our results with some states of artworks, and found that our experimental results show a high capacity and performance of different sub-regions of the tumor. Future work will focus on improving these results, especially enhancing tumors and adopting our method for the 3D segmentation of brain tumors.

## Figures and Tables

**Table 1:** The main layers’ output size of our proposed architecture.

Layer	Output Size
Input	(224,224,3)
VGG-19 + BiFPN ^1^	[(224,224,32), (112,112,32), (56,56,32), (28,28,32), (14,14,32)]
MobileNetV2 + BiFPN ^2^	[(224,224,32), (112,112,32), (56,56,32), (28,28,32), (14,14,32)]
ResNet50 + BiFPN ^3^	[(224,224,32), (112,112,32), (56,56,32), (28,28,32), (14,14,32)]
Fusion of 1, 2, and 3	[(224,224,32), (112,112,32), (56,56,32), (28,28,32), (14,14,32)]
Attention decoder	(224,224,128)
Output	(224,224,4)

^1^ Encoder1, ^2^ Encoder2, ^3^ Encoder3.

**Table 2:** Metrics of our method on the BraTS 2020 training dataset.

Subset		DSC (%)			IoU (%)			HD95(mm)	
	WT	TC	EnT	WT	TC	EnT	WT	TC	EnT
**Training**	92.11	90.30	88.78	82.25	78.90	76.55	0.0	1.00	0
**Validation**	87.89	76.45	67.63	78.39	61.88	51.09	0.0	2.00	2.84
**Test**	87.41	80.69	70.33	77.64	67.63	54.24	0.0	1.00	0.0

“WT” means “whole tumor ”,”TC” means “core tumor”, “EnT” means “enhancing tumor”.

**Table 3:** Precision, F1-score, recall, and accuracy of our method on the BraTS 2020 training dataset.

Subset	Precision (%)			F1-Score (%)			Recall (%)			Accuracy (%)		
	WT	TC	EnT	WT	TC	EnT	WT	TC	EnT	WT	TC	EnT
**Training**	91.19	90.65	88.41	90.5	89.13	86.19	90.08	88.77	84.06	99.90	99.45	98.79
**Validation**	87.50	85.34	83.66	87.3	84.91	82.44	86.22	84.04	82.15	99.20	98.10	97.15
**Test**	90.40	89.79	86.23	88.9	88.54	83.93	87.88	87.04	82.98	99.77	99.23	98.30

**Table 4:** Performance comparison between our proposed method and different supervised and non-supervised approaches on different BRATS datasets.

Method	Data		Performance of (%)	
		WT	TC	EnT
Bisecting (no initialization) [39]	MRI collected by authors	ACC 83.05	-	-
Aboussaleh et al. [14]	BraTS2017	DSC 82.35	-	-
Wu et al. [25]	BraTS2018	DSC (avg) 61.90	-	-
K-means and FCM [3]	https://radiopaedia.org/ (accessed on 1 September 2022)	ACC 56.40	-	-
U-Net [15]	BraTS2020	DSC 80	DSC 62	DSC 60
U-Net-VGG16 [18]	Data approved by Dr. Soetomo Surabaya	ACC (avg) 96.10	-	-
Single path MLDeepMedic [42]	BraTS2017	DSC 79.73	DSC 71.59	DSC 68.14
Fang et al. [40]	BraTS2018	DSC 85.60	DSC 72.20	DSC 72.60
Chen et al. [41]	BraTS2018	DSC 83.60	DSC 68.90	DSC 78.30
HTTU-Net [17]	BraTS2018	DSC 86.50	DSC 74.50	DSC 80.80
AGResU-Net [24]	BraTS2019	DSC 87	DSC 77.70	DSC 70.90
**Our method**	**BraTS2020**	**DSC 87.41**	**DSC 80.69**	**DSC 70.33**

“-” means “none”, “avg” means “average ”, “ACC” means “accuracy”.

**Table 5:** The proposed method ablation study.

Method		DSC (%)			IoU (%)			HD96 (mm)		Accuracy (%)
	WT	TC	EnT	WT	TC	EnT	WT	TC	EnT	AVG
**U-Net**	80.0	62.0	60.0	67.0	52.0	42.0	2.84	2.0	2.83	92.0
**VGG-19 + Decoder**	81.32	75.13	61.66	68.53	60.17	44.57	2.0	2.00	0.0	97.26
**MobileNetV2 + Decoder**	86.43	80.51	71.25	76.11	67.44	55.34	1.0	1.00	0.0	97.0
**ResNet50 + Decoder**	85.78	78.77	70.51	75.09	64.97	54.45	2.0	1.13	0.0	97.0
**3Encoder + Decoder**	84.77	77.13	67.01	73.57	62.77	50.39	2.0	1.41	**0.0**	98.29
**3Encoder + AttDecoder**	79.16	72.89	61.92	62.86	55.86	45.07	3.0	2.00	**0.0**	97.11
**3Encoder + BiFPN + Decoder**	86.88	80.55	70.11	76.58	67.51	54.11	0.0	1.00	0.0	98.99
**3Encoder + BiFPN+ AttDecoder (our method)**	**87.41**	**80.69**	**70.33**	**77.64**	**67.63**	**54.24**	**0.0**	**1.0**	**0.0**	**99.10**

“AVG” means “average”.

## Data Availability

We use open data from Kaggle BraTS2020. The link is https://www.kaggle.com/datasets/awsaf49/brats20-dataset-training-validation (accessed on 1 January 2022).
